# Allowing for missing outcome data and incomplete uptake of randomised interventions, with application to an Internet-based alcohol trial

**DOI:** 10.1002/sim.4360

**Published:** 2011-09-21

**Authors:** Ian R White, Eleftheria Kalaitzaki, Simon G Thompson

**Affiliations:** a Medical Research Council Biostatistics UnitCambridge, U.K.; b Medical Research Council General Practice Research FrameworkLondon, U.K.

**Keywords:** missing data, missing not at random, non-compliance, randomised trials

## Abstract

Missing outcome data and incomplete uptake of randomised interventions are common problems, which complicate the analysis and interpretation of randomised controlled trials, and are rarely addressed well in practice. To promote the implementation of recent methodological developments, we describe sequences of randomisation-based analyses that can be used to explore both issues. We illustrate these in an Internet-based trial evaluating the use of a new interactive website for those seeking help to reduce their alcohol consumption, in which the primary outcome was available for less than half of the participants and uptake of the intervention was limited.

For missing outcome data, we first employ data on intermediate outcomes and intervention use to make a missing at random assumption more plausible, with analyses based on general estimating equations, mixed models and multiple imputation. We then use data on the ease of obtaining outcome data and sensitivity analyses to explore departures from the missing at random assumption. For incomplete uptake of randomised interventions, we estimate structural mean models by using instrumental variable methods.

In the alcohol trial, there is no evidence of benefit unless rather extreme assumptions are made about the missing data nor an important benefit in more extensive users of the intervention. These findings considerably aid the interpretation of the trial's results. More generally, the analyses proposed are applicable to many trials with missing outcome data or incomplete intervention uptake. To facilitate use by others, Stata code is provided for all methods. Copyright © 2011 John Wiley & Sons, Ltd.

## 1. Introduction

Missing outcome data and incomplete uptake of trial interventions are common problems in randomised controlled trials. A key consideration in handling both issues is the intention-to-treat (ITT) principle [1], which states that all individuals randomised in a clinical trial should be included in the analysis, in the groups to which they were randomised, regardless of any departures from randomised treatment. Following this principle preserves the benefit of randomisation, that the treatment groups cannot differ systematically on any factors except those assigned in the trial, and avoids selection bias. However, it is not universally agreed how the ITT principle applies when some outcomes are missing [2]. Further, the ITT principle does not tell us how to estimate the effects that might have been observed with better uptake of trial interventions.

Missing outcome data are problematic because they cause a loss of power and can lead to biased estimates of intervention effects. Once data are missing, the loss of power cannot be reversed, but it can be minimised by appropriate analysis choices, in particular by including all observed data in the analysis [3]. Estimates of intervention effects are typically biased if the analysis makes the wrong assumption about the missing data. However, any analysis with missing data must make partly or completely untestable assumptions, so we can rarely be sure that we have the correct analysis. For this reason, sensitivity analysis is recommended [4–6].

The assumptions of many (but not all) statistical methods for handling missing data can be expressed using the framework of Little and Rubin [7]. Data are missing completely at random (MCAR) if the probability of data being missing does not depend on any missing or observed values. Data are missing at random (MAR) if the probability of a particular set of values being missing for an individual does not depend on the values themselves, conditional on the observed values of other variables. Otherwise, data are missing not at random (MNAR).

Incomplete uptake of trial interventions often means that randomised groups have more similar experience than the investigators had intended, which usually causes the difference in outcomes to be smaller than it would have been with better uptake [8]. However, bias in the estimated intervention effect is not always towards zero: incomplete uptake in equivalence or non-inferiority trials, or in trials where non-trial interventions are available, can inflate differences between randomised groups [9]. Estimating the effect of *allocating* an intervention does not require adjustment for incomplete uptake, in contrast to estimating the effect of a particular level of intervention uptake. The latter is commonly carried out by per-protocol analysis, which excludes data observed when participants had poor intervention uptake. However, per-protocol analysis is undesirable because it is subject to selection bias. Randomisation-respecting alternatives achieve the same aim by using only comparisons of groups as randomised [8]. One such method is principal stratification [10], which leads to estimation of the complier-average causal effect (CACE) [11] in problems where intervention uptake is dichotomous. An alternative, suitable for quantitative intervention uptake, is the structural mean model (SMM) [12].

This paper aims to promote the implementation of recent methodological developments by describing a sequence of analyses that explores both issues and to illustrate the methods using data from an Internet-based trial. This trial is a good example because the issues are particularly acute, but they arise in a wide range of other trials. The Internet-based trial is described in Section 2. Methods for tackling missing data are described in Section 3, with results in Section 4. Methods for tackling incomplete uptake of interventions are described in Section 5, with results in Section 6. We conclude with a discussion in Section 7.

## 2. The Down Your Drink trial

Hazardous drinking in the general population is an important public health problem [13]. Brief interventions are effective [14] but hard to implement. The Internet is increasingly used to deliver behaviour change interventions [15], and a new ‘Down Your Drink’ (DYD) website was developed, building on psychological theories and aiming to engage users by providing interactive tools [16].

The DYD trial was a randomised evaluation of the DYD website compared with a non-interactive control website providing information only [17]. All stages of the trial—recruitment, randomisation, intervention and data collection—were conducted online. This presented a number of challenges [18]. The key challenge relevant to the present paper was whether the numbers of participants using the intervention website and providing follow-up data would be sufficient.

The primary trial outcome was alcohol consumption in the previous week, which was recorded by the TOT-AL, a specially developed online questionnaire [19]. When an outcome assessment was due, participants received an email with a link to the trial website where they could complete the outcome questionnaires. Alcohol consumption was transformed in all analyses to log(number of units in the last week plus 1).

This paper uses data, summarised in Table I, from the pilot trial, which recruited 3746 individuals from 16 February to 16 October 2007. Outcome data were collected at 1 and 3 months; we focus on estimating the intervention effect at 3 months. The correlation between baseline and 3-month alcohol consumption was 0.41 (0.45 for baseline and 1 month; 0.53 for 1 month and 3 months).

**Table I:** Down Your Drink trial: data description

		Intervention (*n* = 1880)		Control (*n* = 1866)	
Baseline variables					
Age (years)		37.5	(10.9)	37.5	(10.9)
Male		45%		44%	
Has degree		51%		50%	
AUDIT-C score (0 to 12)		8.4	(2.1)	8.4	(2.1)
EQ-5D score ( −0.6 to 1)		0.85	(0.18)	0.85	(0.18)
Confidence score (1 to 5)		2.8	(1.2)	2.8	(1.2)
TOT-AL (units/week)		56.0	(36.8)	54.7	(37.3)
log(TOT-AL + 1)		3.80	(0.82)	3.77	(0.84)
Compliance variables					
No. of logins in the first month:	0	5%		5%	
	1	59%		72%	
	≥ 2	36%		23%	
No. of pages hit in the first month		64.9	(78.6)	12.7	(12.8)
Complier at 1 month		78%		—	
No. of logins in the first 3 months:	0	4%		5%	
	1	51%		60%	
	≥ 2	45%		35%	
No. of pages hit in the first 3 months		70.4	(89.7)	13.7	(13.9)
1-month outcome variables					
Responded		50%		60%	
TOT-AL (units/week)		39.8	(34.0)	39.5	(32.8)
log(TOT-AL + 1)		3.30	(1.10)	3.30	(1.10)
3-month outcome variables					
Responded		38%		46%	
TOT-AL (units/week)		38.6	(32.6)	37.0	(32.5)
log(TOT-AL + 1)		3.25	(1.12)	3.18	(1.18)

Values are arithmetic mean (SD) or %. AUDIT-C, Alcohol Use Disorders Identification Test-C; EQ-5D, EuroQol Five Dimensional.

Although baseline data were complete, poor follow-up response rates were anticipated because there was no personal contact with participants. To increase response rates, all participants who did not complete the outcome questionnaires within 7 days of the first email invitation received second and (if necessary) third invitations at weekly intervals. A fourth email inviting participants to provide their outcome data directly by email to the investigators yielded no further responses. Offline follow-up was attempted for users who had provided a telephone number or address, but was not successful [18]. Incentives were also trialled [20]. Participants were additionally randomised to complete only one of four secondary outcome measures in order to reduce the assessment burden and improve response rates [21]. The number of emails sent to each participant is summarised in Table II and is used in the analysis in Section 4. In the intervention arm, the mean log(TOT-AL + 1) is larger in later respondents than earlier respondents, suggesting a MNAR mechanism, with non-respondents perhaps having an even higher mean log(TOT-AL + 1).

**Table II:** Down Your Drink trial: outcome data at 3 months by number of emails sent

	Intervention				Control			
		
			log(TOT-AL + 1)				log(TOT-AL + 1)	
						
Responded after email	*n*	%	Mean	SD	*n*	%	Mean	SD
1	348	19	3.16	1.16	441	24	3.17	1.19
2	194	10	3.28	1.04	236	13	3.18	1.19
3	174	9	3.41	1.11	178	10	3.22	1.17
Never	1164	62	—	—	1011	54	—	—
Total	1880	100	3.25	1.12	1866	100	3.18	1.18

Similarly, low use of the website was a concern. It is hard to define and measure website use [22]; in particular, although each page download was recorded, the length of time that participants spent actually using the website is unknown. We summarised website use by the number of login sessions and the total number of pages downloaded in the first month; in calculating the latter, multiple downloads of the same page were counted only if they occurred in different login sessions. Individuals were automatically logged in to the intervention or control websites after randomisation, but a few who immediately left the trial website had no logins.

The main findings of the trial were that alcohol consumption in responders dropped substantially from baseline to 1 month, and again slightly from 1 to 3 months, but that the drops were very similar across randomised groups [23]. The ratio of (geometric mean) 3-month alcohol consumption in the intervention group compared with the control group was 1.04 (95% confidence interval 0.94 to 1.16). However, missing data were substantial and more common in the intervention group (Table I). Use of the intervention website was greater than use of the control website, but the majority of participants in both arms had only one login session.

The outstanding questions that this paper aims to answer are whether the results are robust to different assumptions about the missing data and whether interpretation is affected by incomplete use of the website.

## 3. Missing outcome data: methods

We propose a modelling strategy that starts with simple data on baseline and outcome and then progressively adds in intermediate outcomes, website use and ease-of-contact data.

For the *i*th participant, let *z*_*i*_ denote their randomised group, ***x***_*i*_ a vector of baseline covariates (assumed complete), *y*_*i*1_, *y*_*i*2_ the outcomes at two follow-up times and *r*_*i*1_, *r*_*i*2_ whether each outcome was observed (1) or missing (0). Our methods generalise easily to more than two follow-up times. If the data were complete, then an adjusted analysis for the outcome at follow-up time 2 would estimate *β* in the model


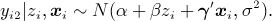
(1)

An unadjusted analysis is the same without the ***γ***^′^***x***_*i*_ term.

### 3.1. Complete cases

A first analysis fits model (1) in the subset with *r*_*i*2_ = 1, the ‘complete cases’. This analysis is inefficient because it does not make use of individuals with *y*_*i*2_ missing but *y*_*i*1_ observed. It is valid if the model is correctly specified and the data are ‘covariate-dependent missing completely at random’ [24]: that is, if the missing data mechanism depends only on the baseline covariates included in the model. If the model is incorrectly specified (e.g. if it should contain a nonlinear function of ***x***_*i*_), then the analysis is in general valid only if the data are MCAR within randomised groups [25].

### 3.2. Using repeated outcome measures

We now consider three methods that jointly model both *y*_*i*1_ and *y*_*i*2_. These are valid if the data (*y*_*i*1_,*y*_*i*2_) are MAR given (*z*_*i*_,***x***_*i*_): in particular, for participants with *y*_*i*1_ observed, dropout at follow-up time 2 is now allowed to depend on *y*_*i*1_.

A *generalised estimating equations* (GEE) approach [26] fits the model:


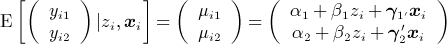
(2)


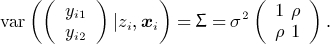
(3)

Normality is not assumed, but the residual variance *σ*^2^ is assumed to be equal at the two times. Estimation uses the standard estimating equations [26]; the parameter of main interest is *β*_2_. If the model is misspecified (in particular, if the residual variance is different at the two times), then valid standard errors can still be obtained by the robust (sandwich) method. With incomplete data, point estimates for a correctly specified model are valid if the data are MAR, whereas point estimates for an incorrectly specified model are valid if the data are MCAR; weighted estimating equations can relax the latter condition to MAR [27]. We allow the coefficients in the two components of (2) to be different: this amounts to allowing interactions between time and the baseline variables ***x*** and *z*. In general, it is best to use an unstructured working correlation matrix; with only two time points, this is the same as an exchangeable working correlation matrix.

A *mixed models* approach [28] modifies the model defined by (2) and (3) by adding the distributional assumption


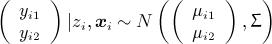
(4)

and replacing (3) with an unconstrained variance–covariance matrix Σ. The model is estimated using restricted maximum likelihood. It may be appropriate to allow Σ to differ by randomised group.

In *multiple imputation* (MI), several completed data sets are produced by drawing the missing values from their posterior predictive distribution thus acknowledging the uncertainty due to missing data under a MAR assumption [29, 30]. This can be carried out using model (4). It is often sensible to draw imputations separately for each trial arm, because interactions between randomised group and baseline covariates may be of interest [31]. It is sometimes considered that MI offers a way to include all randomised individuals in the analysis (e.g. [32]). However, if the imputation model is the same as the analysis model, then MI is expected to give approximately the same results as a mixed model analysis [33].

### 3.3. Using compliance

One way to make the MAR assumption more plausible is to introduce other post-randomisation variables ***v***_*i*_ into the analysis. Specifically, we now assume that (*y*_*i*1_,*y*_*i*2_,***v***_*i*_) are MAR given (*z*_*i*_,***x***_*i*_), or if ***v***_*i*_ is complete that (*y*_*i*1_,*y*_*i*2_) are MAR given (*z*_*i*_,***x***_*i*_,***v***_*i*_), so that observed values of ***v***_*i*_ are allowed to explain missingness of (*y*_*i*1_,*y*_*i*2_). Here, we take ***v***_*i*_ as the amount of intervention received (compliance), because this is likely to predict both outcomes (*y*_*i*1_,*y*_*i*2_) and responses (*r*_*i*1_,*r*_*i*2_), but ***v***_*i*_ could also include trial outcomes that are more observed than *y*_*i*2_.

In the mixed model approach, ***v***_***i***_ can be included using the extended model [5]


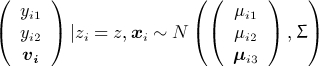
(5)

where *μ*_*i*1_ and *μ*_*i*2_ are still as defined in (2) and 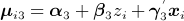. Σ is modelled completely flexibly. The GEE approach is similar but without the normality assumption; Σ is modelled with an unstructured working correlation and equal variances, so it is advisable to scale the compliance variables to have variances similar to the outcome variables. Including ***v***_***i***_ is easiest under the MI approach, because it can simply be included in the imputation model and excluded from the analysis model.

In many trials, compliance has a very different distribution across the two arms and may have different meaning. In this case, it is important to allow the association between ***v***_*i*_ and (*y*_*i*1_,*y*_*i*2_) to vary by randomised group. This is most conveniently carried out in MI, by imputing separately by arm; it cannot be carried out using standard GEE implementations, but a mixed model could allow Σ in (5) to depend on *z*_*i*_.

### 3.4. Sensitivity analyses

The aforementioned models attempt to make a MAR assumption more plausible by including more data in the analysis [34]. However, MAR often remains at least questionable, if not implausible [35]. We now consider sensitivity analyses to departures from MAR. Following Kenward *et al.* [4], we embed the MAR model in a wider family of MNAR models indexed by one or more ‘informative missing parameters’ that express the magnitude of departures from MAR. We then use subject-matter knowledge to specify possible values of the informative missing parameters and re-estimate the intervention effect in each case.

We use a pattern-mixture model [36] that extends Equation (1) by allowing a term *δ*^*Y*^ that controls departures from MAR:


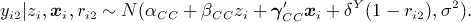
(6)

The regression parameters subscripted CC can be estimated by fitting Equation (1) to the complete cases (*r*_*i*2_ = 1), but *δ*^*Y*^ is not identified by the data. An important extension allows the informative missing parameter *δ*^*Y*^ to differ between randomised groups:


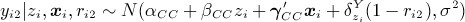
(7)

where 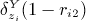 can also be written as 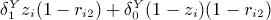. This model is plausible because, for example, missing data may well be more informative among individuals who have been encouraged to change their behaviour than among controls, and is important because treatment effects are most affected when departures from MAR behave differently in the two arms [37]. Parameters 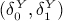 are not identified by the data: 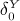 is the mean difference between unobserved and observed outcomes in the control arm, adjusted for ***x***, and 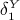 is the corresponding difference in the intervention arm.

This model has previously been used with an informative prior distribution for 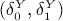 that was elicited from investigators [37]. In the present paper, investigators’ views are used to define plausible values of 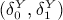 for sensitivity analysis, rather than tackling a fully Bayesian analysis. It is useful to consider three sensitivity analyses: first, to values of 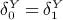, then to values of 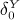 fixing 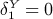 and, finally, to values of 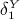 fixing 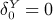.

Once 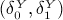 has been specified, estimation is straightforward. Write 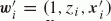 and 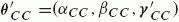 so the mean part of model (6) is 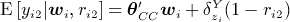. The model of interest is E [*y*_*i*2_ | ***w***_*i*_] = ***θ***^′^***w***_*i*_ where ***θ***^′^ = (*α*,*β*,***γ***^′^). It follows that ***θ*** = ***θ***_*CC*_ +***θ***_*ADD*_ where ***θ***_*ADD*_ are the coefficients from a regression of 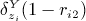. It is easy to estimate ***θ***_*CC*_ and ***θ***_*ADD*_. Finally, the estimated parameters are independent, so we can estimate 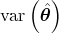 as 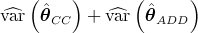.

### 3.5. Using the number of attempts

Instead of specifying the informative missing parameter(s) based on subject-matter knowledge, it may be possible to estimate them using data on the number of attempts made to observe outcome *y*_*i*2_. Let *r*_*i*2*k*_ be the outcome of the *k*th attempt to observe the primary outcome *y*_*i*2_, where *r*_*i*2*k*_ = 1 indicates that the outcome was observed, *r*_*i*2*k*_ = 0 indicates that it was not observed and *r*_*i*2*k*_ = ⋅ indicates that the *k*th attempt was not made (either because a previous attempt was successful or because the participant had refused or withdrawn from the trial). The association between *r*_*i*2*k*_ and *y*_*i*2_ can be identified using Alho's model, which assumes that this association is the same for all *k* [38]:


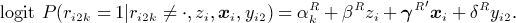
(8)

Here, we allow the probability of responding to vary between attempts, but we assume that the association between fully observed covariates and responding is the same at all attempts, although the latter assumption could easily be relaxed. *δ*^*R*^ is an informative missing parameter, and *δ*^*R*^ = 0 corresponds to MAR.

Estimation of model (8) uses data on individuals with observed outcomes together with the numbers and baseline covariates of individuals with unobserved outcomes. The model may be fitted using a conditional likelihood supplemented by a set of estimating equations [38], but this algorithm is not guaranteed to converge. Alternative estimation methods are based on the full likelihood for model (8) jointly with model (4). A Bayesian approach has been used [39], and a likelihood-based approach is also possible; the likelihood involves integrating out the unobserved values of *y*_*i*2_. Fitting the model by using the full likelihood directly estimates the parameters of (4); an alternative is to use the inverse of the response probability as a weight for analysis of complete cases [38], but care must be taken to obtain standard errors that allow for the often large uncertainty in the weights [39].

As in the previous section, an important extension to model (8) allows the informative missing parameter *δ*^*R*^ to differ between randomised groups:


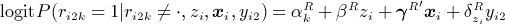
(9)

where 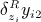 can also be written as 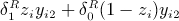.

## 4. Missing outcome data: analysis of the Down Your Drink trial

### 4.1. Implementation

In the DYD trial, *z*_*i*_ = 1 for individuals randomised to the interactive website and 0 for the control website, and (*y*_*i*1_,*y*_*i*2_) are the 1-month and 3-month alcohol consumption outcomes (log(TOT-AL + 1)). All analyses were performed both unadjusted and adjusted for ***x***_*i*_, which comprises the baseline variables listed in Table I; in general, we prefer the analysis adjusted for baseline covariates, especially the baseline value of the outcome. For GEEs, robust standard errors were used. For mixed models, the unstructured variance–covariance matrix was allowed to differ between randomised groups, although results with a common variance–covariance matrix (not shown) were very similar.

Multiple imputations were drawn separately for each arm by using the chained equations approach [40] implemented in Stata [41, 42]. For method MI1, the imputation model for the outcome at each time was a linear regression including the outcome at the other time and the baseline variables. For method MI2, the imputation models additionally included the log of one plus the numbers of pages hit at 1 and 3 months and the log of one plus the numbers of login sessions at 1 and 3 months. The distribution of the incomplete variables was not fully Normal, even after log transformation, so predictive mean matching was used to improve the imputations [31]. In each case, model (1) was fitted to each of 50 imputed data sets, and the results were combined using Rubin's rules [29]. Covariates were used in the imputation model even when unadjusted analyses were performed.

For sensitivity analyses, the views of five DYD investigators were quantified before the trial results were known, and these views were used to choose values of the informative missing parameters 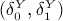 in Equation (7). When the informative missing parameters were assumed the same in both arms, the investigators believed that the mean of the unobserved responses for alcohol consumption at 3 months could be as much as 75% more or 50% less than the mean of the observed responses: these suggest the sensitivity analyses 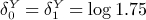 and 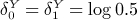. When the data were assumed to be informatively missing only in the control arm, the investigators believed that the mean of the unobserved responses could be as much as 50% more or 50% less than the mean of the observed responses: these suggest the sensitivity analyses 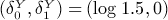 and (log 0.5,0). We also choose the corresponding cases with the data informatively missing only in the intervention arm: 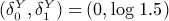 and (0, log 0.5). In addition to these rather extreme sensitivity analyses, we also used more moderate sensitivity analyses with 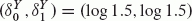, (log 1.25,0) and (0,log 1.25).

Analysis of number of attempts used the number of email reminders that were sent to each participant. The conditional likelihood algorithm diverged in some cases, so we used the maximum likelihood approach. ‘Alho 1’ and ‘Alho 2’ refer to models (8) and (9), respectively.

Stata code for these analyses is given in Appendix A.1.

### 4.2. Results

**Figure 1:**
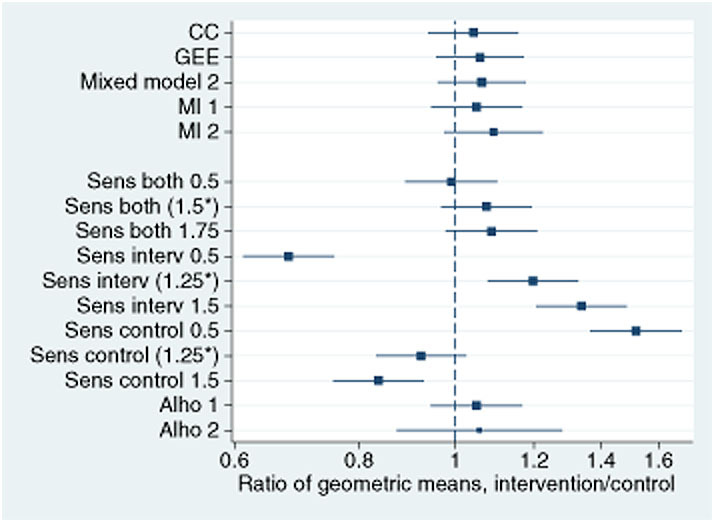
Down Your Drink trial: estimates (95% confidence intervals) of the intervention effect on weekly alcoholconsumption, adjusted for baseline covariates, using different methods for handling the missing data. *Denotesmoderate sensitivity analyses.

We summarise the results in Table III and display the covariate-adjusted results in Figure 1. The intervention effect is expressed as the ratio of the geometric mean alcohol consumption (plus 1 unit/week) at 3 months in the intervention group to the corresponding geometric mean in the control group. In the following text, we interpret these figures as percentage increases or decreases.

**Table III:** Down Your Drink trial: analysis of alcohol consumption at 3 months, using various assumptions and methodsto handle missing outcome data

Approach	Assumption	Method	Unadjusted	Adjusted
Complete cases				
	CD-MCAR^1^		1.073 (0.956 to 1.203)	1.043 (0.939 to 1.157)
Using repeated outcome measures				
	MAR^2^	GEE	1.095 (0.982 to 1.222)	1.058 (0.957 to 1.171)
		Mixed model	1.097 (0.983 to 1.223)	1.063 (0.960 to 1.176)
		MI1	1.072 (0.960 to 1.196)	1.050 (0.945 to 1.167)
Using website use				
	MAR^3^	MI2	1.115 (0.991 to 1.255)	1.092 (0.974 to 1.225)
Sensitivity analysis using (7)		exp (*δ*)		
	MNAR, 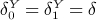	0.5	1.017 (0.905 to 1.142)	0.990 (0.890 to 1.101)
		1.5*	1.107 (0.986 to 1.242)	1.074 (0.967 to 1.193)
		1.75	1.120 (0.997 to 1.258)	1.087 (0.978 to 1.208)
	MNAR, 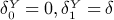	0.5	0.698 (0.622 to 0.784)	0.680 (0.612 to 0.755)
		1.25*	1.232 (1.098 to 1.381)	1.197 (1.078 to 1.328)
		1.5	1.379 (1.229 to 1.547)	1.339 (1.206 to 1.487)
	MNAR, 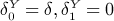	0.5	1.562 (1.391 to 1.753)	1.519 (1.367 to 1.688)
		1.25*	0.951 (0.847 to 1.066)	0.924 (0.832 to 1.025)
		1.5	0.861 (0.768 to 0.966)	0.837 (0.753 to 0.929)
Using the number of attempts and (9)				
	MNAR, 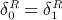	Alho 1	1.086 (0.967 to 1.220)	1.050 (0.945 to 1.167)
	MNAR, 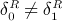	Alho 2	1.328 (0.926 to 1.904)	1.057 (0.872 to 1.281)

Figures are ratio of geometric means, intervention/control, with 95% confidence interval.

* Moderate sensitivity analyses.

1 *y*_*i*2_ is covariate-dependent MCAR given (*z*_*i*_,***x***_*i*_).

2 (*y*_*i*1_,*y*_*i*2_) are MAR given (*z*_*i*_,***x***_*i*_).

3 (*y*_*i*1_,*y*_*i*2_) are MAR given (*z*_*i*_,***x***_*i*_,***v***_*i*_).

All methods based on MAR, as well as complete-cases analysis, give very similar results: the point estimate represents a non-significant increase of between 4% and 12% due to the intervention, with a 95% confidence interval that does not extend below a 6% reduction.

Sensitivity analyses show that the estimated intervention effect is not very sensitive to departures from MAR when the informative missing parameter is assumed to be equal across randomised groups, but is very sensitive to departures from MAR that occur differently in the randomised groups. Moderate sensitivity analyses (indicated by * in Table III and Figure 1) yield estimates ranging from an 8% reduction to a 23% increase in alcohol consumption, whereas more extreme sensitivity analyses range from a 32% reduction to a 56% increase. This suggests that the trial's results are only robust to departures from MAR that are similar in both randomised groups.

Using the number of email reminders and the MNAR models (8) and (9) gives the estimates of the informative missing parameters in Table 4. For model (8), where the informative missing parameter is assumed equal across the two groups, the negative estimate of the informative missing parameter *δ*^*R*^ suggests that heavier drinkers are more likely to be non-responders. However, the informative missing parameter is not significantly different from zero so that the data are consistent with a MAR assumption. For model (9), where the informative missing parameter is allowed to differ between groups, both estimates are again negative and that for the intervention group is larger in magnitude, suggesting that the tendency for heavier drinkers to be non-responders may be greater in the intervention group. Although the informative missing parameter in the intervention group is significantly different from zero (*P* = 0.03), a test for a difference between the arm-specific informative missing parameters is not significant (*P* = 0.26) nor is a test on 2 degrees of freedom for departure from MAR (*P* = 0.09).

**Table IV:** Down Your Drink trial: informative missing parameters *δ*^*R*^(standard errors), defined as the log odds ratio for response per 1-unit increase in 3-month log(TOT-AL + 1),estimated using the Alho model and maximum likelihood

Model	Arm	Unadjusted	Adjusted
Alho 1	Both	−0.15 (0.10)	−0.10 (0.13)
Alho 2	Intervention	−0.28 (0.13)	−0.11 (0.16)
	Control	−0.06 (0.15)	−0.10 (0.13)

Alho 1: MNAR, common *δ*^*R*^ across attempts and arms (model (8)).

Alho 2: MNAR, common *δ*^*R*^ across attempts (model (9)).

These results do not provide good evidence against a MAR assumption, but they change the estimated intervention effects in Table III when the informative missing parameter is allowed to differ across randomised groups as in model (9). This MNAR analysis indicates a much larger increase due to intervention in the unadjusted analysis, and much wider confidence intervals in both unadjusted and adjusted analyses. We attribute these findings to the great sensitivity of estimated intervention effects to differences in informative missing parameter *δ*^*R*^ between randomised groups, along with the difficulty of estimating this parameter.

## 5. Incomplete uptake of interventions: methods

Intervention receipt in some randomised trials can be summarised as a binary variable [43], whereas other trials have complex intervention receipt that may be summarised as one or more quantitative variables. We present a SMM that is applicable to both binary and quantitative cases, provided that intervention receipt is univariate.

### 5.1. Structural mean model

Structural mean models describe the relationship between the observed data and the counterfactual data that would have been observed with a different random allocation [12, 44]. For the *i*th individual, we define *y*_*i*_(1) as the outcome that would be observed if they were randomised to intervention and *y*_*i*_(0) as the corresponding outcome if they were randomised to control. Exactly one of these potential outcomes is observed for each individual. Define *d*_*i*_(1) as the *i*th individual's compliance (binary or quantitative) with the intervention, if they were allocated to intervention. We initially ignore compliance with the control. We now assume that the causal effect of the intervention is proportional to the compliance. This implies that individuals who would be complete non-compliers if allocated to intervention have no effect of allocation, the ‘exclusion restriction’ assumption. We then have the SMM


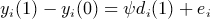
(10)

where *e*_*i*_ is a zero-mean error term whose presence allows treatment effects to vary between individuals. Model (10) implies that


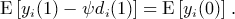
(11)

Estimation proceeds by noting that randomised group *z*_*i*_ is independent of the potential outcomes *y*_*i*_(1), *y*_*i*_(0) and *d*_*i*_(1), so each expectation in (11) can be computed in one arm of the trial. This leads to the estimating equation 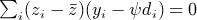 where *d*_*i*_ = *d*_*i*_(1) or 0 for individuals randomised to intervention or control, respectively.

Baseline covariates ***x***_*i*_ that are uncorrelated with *z*_*i*_ and *e*_*i*_ may be used in two ways to improve the efficiency of the estimation procedure. First, we can condition on ***x***_*i*_ in (11) and model E [*y*_*i*_(0) | ***x***_*i*_] = *α*+***γx***_*i*_, yielding the alternative estimating equation 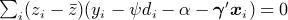, to which we add standard estimating equations for *α* and ***γ*** [45], giving


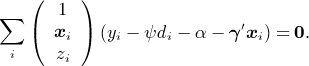
(12)

Equation (12) is easy to estimate because it is a standard instrumental variables (IV) model [46], in which *z*_*i*_ is the instrument, *d*_*i*_ is the ‘endogenous’ variable and ***x***_*i*_ is the ‘exogenous’ variable. A second approach, not adopted here, makes use of baseline covariates ***w***_*i*_ that predict *d*_*i*_ in the intervention group. In this case, precision can be gained by using the interactions *z*_*i*_***w***_*i*_ as additional instruments, but at the cost of further assumptions [47]. Again, this can be fitted using standard IV software.

### 5.2. Interpretation

Interpreting the estimated parameter *ψ* is easiest when compliance is binary. In the aforementioned model, this means that *d*_*i*_(1) is 0 or 1. In the statistical literature, the two groups formed are often known as ‘compliers’ (*d*_*i*_(1) = 1) and ‘non-compliers’ (*d*_*i*_(1) = 0), referring to an individual's compliance status if they were randomised to the intervention. In this setting, the ‘exclusion restriction’ assumption, which identifies the model, states that randomised allocation has no effect on non-compliers. The parameter *ψ* can be interpreted without further assumptions as the CACE, the average of *y*_*i*_(1) −*y*_*i*_(0) over the subgroup with *d*_*i*_(1) = 1 [48].

If compliance is not naturally binary, it is tempting to dichotomise it. Clearly, a good definition of ‘compliers’ is needed. It is not necessary to assume that compliers all receive the same benefit of intervention, because the CACE represents an average overall compliers. However, it is essential to assume that non-compliers receive no benefit from intervention. It is therefore typically necessary to use a restrictive definition of non-compliance, classing any individual whose moderate compliance could have brought him or her benefit with the compliers.

An alternative approach is to express compliance *d*_*i*_(1) quantitatively. This typically makes the exclusion restriction more plausible, because the zero level can be chosen to represent no use of the intervention. However, without further assumptions, no simple interpretation of *ψ* generalises the CACE. The further assumption usually made is E [*e*_*i*_ | *d*_*i*_] = 0 so that model (10) is correctly specified. In this case, *ψd* can be interpreted as the average causal effect of allocation to intervention in the subgroup who would comply to an extent *d*: that is, E [*y*_*i*_(1) −*y*_*i*_(0) | *d*_*i*_(1) = *d*] = *ψd*.

### 5.3. Using control group compliance

When the control group also receives some intervention, such as a placebo or a standard treatment, control-group compliance *d*_*i*_(0) is also available. The SMM could then be extended as


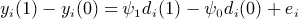
(13)

which allows for a causal effect of the control intervention. The original approach to this problem assumed that *d*_*i*_(1) is a monotonic function of *d*_*i*_(0) [49], but the method is very sensitive to departures from this assumption [50]. More recent causal estimation methods for models such as (13) use either an assumption that *d*_*i*_(0) ≤ *d*_*i*_(1) for all *i* and Bayesian modelling with slightly informative priors [51], or informative priors for one of the treatment effects [52], or covariates that predict *d*_*i*_(0) and *d*_*i*_(1) differently but that do not modify the causal effect of treatment [45]. Because of the complexities of all these approaches, we would prefer to ignore *d*_*i*_(0) when it is plausible that the control intervention has no causal effect (i.e. that *ψ*_0_ = 0 in (13)).

### 5.4. Missing data

Missing outcome data complicate estimation of the IV model. Standard implementations of IV are restricted to using complete cases only and are thus valid only under MCAR. Three approaches can be used to make them valid under MAR.

First, inverse probability weighting (IPW) can be used [53, 54]. Models are constructed for *p*(*r*_*i*_ | *x*_*i*_,*z*_*i*_ = 1,*d*_*i*_) and *p*(*r*_*i*_ | *x*_*i*_,*z*_*i*_ = 1), and the ‘stabilised weights’ [55] in group 1 are formed as the ratio of the fitted values, 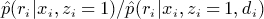. The stabilised weights in group 0 are all 1, because *d*_*i*_ does not vary. Weighted IV regression is then performed with robust standard errors.

Second, the ‘adjusted treatment received’ (ATR) method [56, 57] is equivalent to IV regression for complete data and is valid when outcomes are MAR [54]. In this method, a linear regression model is first constructed for actual treatment receipt on randomised group and covariates (using all observations including those with missing *y*_*i*_), and the residuals are estimated. The causal effect of actual treatment receipt is then estimated by linear regression of *y*_*i*_ on actual treatment receipt, adjusting for the previously estimated residuals and the covariates. The standard errors from this second stage may be underestimated because they ignore uncertainty in the residuals [54].

Third, MI can be used.

## 6. Incomplete uptake of interventions: analysis of the Down Your Drink trial

### 6.1. Implementation

The DYD trial has complex intervention receipt: individuals could use the website on different numbers of occasions, for different lengths of time, and in different ways. Any attempt to estimate the effect of intervention receipt in such data relies on a plausible causal model describing how intervention receipt may affect outcomes. We describe one approach with dichotomised compliance and one with quantitative compliance.

For dichotomised compliance, a non-zero cut-off was chosen, because almost all randomised individuals had at least one login (Table I). Section 5.2 argues for a relatively low cut-off, and we defined compliers as individuals who logged in more than once or accessed more than 10 pages of the website within the first 1 month from randomisation. Our analyses therefore rest on the assumption that an individual who accessed fewer than 10 pages on only one occasion received no benefit, and they estimate the average benefit of the intervention website over a wide range of use.

For quantitative compliance, we defined *d*_*i*_(1) in Equation (10) as the number of pages downloaded over the first month of the trial, but with an upper limit of 300 pages because we did not believe that use above this level would have further benefit. We did not use website uptake in the control group in the model, because the control website is unlikely to be effective. For the MI approach, we used the imputations constructed using compliance variables as in MI2 of Section 4.

Stata code for these analyses is given in Appendix A.2.

### 6.2. Results

Of 1880 individuals allocated to intervention, 1461 (78%) were classed as compliers. As a result, the estimated CACE (Table IV) was not very different from the ITT MAR estimate (Table III). IPW and ATR methods behaved very similarly. MI gave somewhat different results: although this is unexpected, it is consistent with the differences between MI1 and MI2 in Table III.

Estimates of the causal effect per 100 pages downloaded were somewhat larger than the ITT estimates. This appears to be because the mean number of pages downloaded in the intervention group was 65, so the estimated effect of downloading 100 pages was approximately one and a half (100/65) times the ITT effect. The confidence interval for the intervention effect in these analyses does not extend below an 11% reduction.

**Table V:** Down Your Drink trial: analysis of alcohol consumption at 3 months, allowing for incomplete use of website

Missing data method	Unadjusted	Adjusted
Binary compliance: compliers versus non-compliers		
IPW	1.100 (0.950 to 1.273)	1.053 (0.921 to 1.203)
ATR	1.100 (0.949 to 1.275)	1.052 (0.920 to 1.204)
Multiple imputation	1.150 (0.986 to 1.342)	1.121 (0.965 to 1.301)
Continuous compliance: per 100 pages downloaded		
IPW	1.124 (0.937 to 1.349)	1.079 (0.917 to 1.269)
ATR	1.123 (0.935 to 1.348)	1.082 (0.916 to 1.277)
Multiple imputation	1.189 (0.982 to 1.440)	1.151 (0.956 to 1.386)

Figures are ratio of geometric means, intervention/control, with 95% confidence interval. ATR, adjusted treatment received; IPW, inverse probability weighting.

## 7. Discussion

### 7.1. Conclusions for the Down Your Drink trial

A concern with the DYD trial, and many other online trials, is that high rates of non-response and low intervention uptake makes it hard to draw conclusions about the intervention's effectiveness. Our analyses in this paper show that the conclusions were not substantially affected under a range of assumptions about the missing data mechanism, except when we assumed that the informative missing parameter differed between randomised groups. To the extent that the latter assumption may be implausible, our results appear reasonably robust. Similarly, conclusions were not substantially affected when we used causal models to consider the impact of downloading 100 website pages. The latter conclusion depends on a judgement that 100 website pages was a reasonable target for moderately conscientious website use.

Our results therefore provide some support for the use of online trials in general, with two cautions: it is essential to consider the informative missingness parameters differing between randomised groups and to consider how much the observed intervention uptake falls short of what might be hoped for. Analyses allowing for non-response and low intervention uptake are best specified in advance and included in the analysis plan.

### 7.2. Methodological conclusions

These methods are of potential use in all trials and not just online trials. When rates of missing data are low, sensitivity analysis may be enough to demonstrate that missing data are not a problem. In other cases, including intermediate or other outcomes and/or compliance variables in MAR analyses is a useful strategy, although treatment effect estimates may only be changed when the auxiliary variables are strongly associated with outcome [58]. Sensitivity analyses are always helpful but depend on expert consideration of the plausible degree of departure from MAR. Data on number of attempts to obtain data, or more generally ease of contact, are often recorded and should be more widely used in analysis: results from the Alho model (8) or (9) can be a useful way to allow for extra uncertainty due to the possibility of MNAR data without the need to rely on expert opinion. With pressure on journal space, it may be convenient for all these alternative analyses to be included in web appendices. In the primary publication of the DYD trial [23], which was based on more data than those used here, the primary analysis was the adjusted complete-cases analysis, and web appendices presented alternative analyses for the missing data—a partial last observation carried forward (LOCF; see in the next section), MI and sensitivity analyses using (7)—and analyses adjusting for non-compliance.

A particularly relevant question in a trial with a ‘negative’ result is whether this negative result is attributable to incomplete intervention uptake. In this context, it is important to formulate the causal question carefully, defining a parameter such as the CACE or the causal effect of a particular amount of intervention, and then consider the limits of the confidence interval for the parameter.

### 7.3. Other methods

We have not reported here an analysis using LOCF, one of the most widely used techniques [3], which simply replaces missing outcomes with the last observed value. LOCF rests on an assumption that outcomes do not change (on average in each arm) after participants drop out of the study, which is often implausible. In the DYD trial, with its large change in outcome after baseline, LOCF would yield implausibly different imputations for individuals with no post-baseline measurement and those with a 1-month measurement. Because LOCF is widely used, the primary DYD trial publication [23] reported a partial LOCF analysis that carried only post-baseline measurements forward. Unfortunately, usual justifications for LOCF rest not on the plausibility of its assumption but on approximate constancy of observed outcomes, or on an appeal to the ITT principle, or on conservatism: none of these are valid [2].

Our sensitivity analyses were based on data from baseline and follow-up time 2 only. Basing the sensitivity analyses on the mixed model (4) might be preferable but is technically more complicated and is unlikely to make much difference in view of the small differences between complete-cases and MAR-based analyses (Table III). The Alho method could also be extended to allow for the repeated measures: for example, better estimating the informative missing parameter *δ*^*R*^ by assuming it to be constant across follow-up times. Another possible assumption about the missing data is that they are ‘latent ignorable’, meaning that they would be MAR if the potential compliance *d*_*i*_(1) were observed for everyone [59].

### 7.4. Extensions

For binary outcomes, mixed models become more complex, and GEE or MI methods might be preferred. The MNAR methods can be applied equally well, and the exp (*δ*) parameters can be interpreted as informatively missing odds ratios [60, 61]. The SMMs described may still be used to estimate causal risk differences, but if causal odds ratios are wanted then generalised SMMs are needed [62].

Methods used for survival outcomes are typically very different from those that we have described. Here, missing data take the form of censoring, and the non-informative censoring assumption takes the place of the MAR assumption: departures from the non-informative censoring assumption are rarely considered but should be. SMMs are not suitable for survival outcomes, but the structural accelerated failure time model is a general alternative for handling incomplete intervention uptake [63, 64], and hazard-based methods are available for handling all-or-nothing uptake [65].

In the DYD trial, all baseline covariates were complete. Incomplete baseline covariates are simply and efficiently handled by single imputation methods such as imputing the overall or centre-specific mean of the covariate [31]. Such simple methods would be inappropriate for missing outcomes: they are appropriate for missing baselines because baseline covariates are independent of randomised group, and adjustment for baseline covariates is not required for unbiased estimation [66].

Stata do-files to implement the analyses presented in this paper are given in Appendix A.

## APPENDIX A. Stata code

### A.1. Missing data

The succeeding code uses four user-written commands. ice [67] and mim [68] implement MI and are available from the Statistical Software Components (SSC) archive. alho and rctmiss implement the ‘number of attempts’ model and sensitivity analyses, respectively, and are available from the first author's website by typing net from http://www.mrc-bsu.cam.ac.uk/IW_Stata/ in Stata.

The code shows adjusted analyses; for unadjusted analyses, delete the global xvars and global time_xvars commands. The data are assumed to be in a file DYDwide.dta with one record per randomised individual.

//Define global macros for different sets of variables
global yvars logtotal1 logtotal3 // Outcome variables
global dvars lpages1 lpages3 lsession1 lsession3
// Intervention uptake variables (logged)
global xvars logtotal_bs age gender educ_cat confidence_0 AUDITC EQ5D_0
// Baseline covariates
global time_xvars i.time#c.logtotal_bs i.time#c.age i.time#i.gender ///
i.time#i.educ_cat i.time#c.confidence_0 i.time#c.AUDITC i.time#c.EQ5D_0
// Interaction terms
// Complete Case Analysis
use DYDwide, clear
regress logtotal3 group $xvars, eform(ratio)
// Generalised Estimating Equations (GEE)
reshape long logtotal, i(userid) j(time) // One record per time per individual
xtset userid time
gen group1 = group*(time==1)
gen group3 = group*(time==3)
xtgee logtotal i.time group1 group3 $xvars $time_xvars, corr(uns) robust eform
// Mixed Model, different variances by arm
xtmixed logtotal i.time group1 group3 $xvars $time_xvars || userid:,nocons ||, ///
residuals(unstructured,t(time) by(group))
// Multiple Imputation
use DYDwide, clear
ice $yvars $xvars, /// Without website use variables
seed(1234) m(50) by(group) match($yvars) saving(imputed1, replace)
ice $yvars $xvars $dvars, /// With website use variables
seed(1234) m(50) by(group) match($yvars) saving(imputed2, replace)
use imputed1, clear // MI1
mim: regress logtotal3 group $xvars, eform(Ratio)
mim, mcerror eform
use imputed2, clear // MI2
mim: regress logtotal3 group $xvars, eform(Ratio)
mim, mcerror eform
// Sensitivity Analyses
use DYDwide, clear
rctmiss, pmmdelta(ln(0.5)) eform(Ratio): reg logtotal3 group $xvars
rctmiss, pmmdelta(ln(1.5)) eform(Ratio): reg logtotal3 group $xvars
rctmiss, pmmdelta(ln(1.75)) eform(Ratio): reg logtotal3 group $xvars
rctmiss, pmmdelta(ln(0.50)*(group==1)) eform(Ratio): reg logtotal3 group $xvars
rctmiss, pmmdelta(ln(1.25)*(group==1)) eform(Ratio): reg logtotal3 group $xvars
rctmiss, pmmdelta(ln(1.50)*(group==1)) eform(Ratio): reg logtotal3 group $xvars
rctmiss, pmmdelta(ln(0.50)*(group==0)) eform(Ratio): reg logtotal3 group $xvars
rctmiss, pmmdelta(ln(1.25)*(group==0)) eform(Ratio): reg logtotal3 group $xvars
rctmiss, pmmdelta(ln(1.50)*(group==0)) eform(Ratio): reg logtotal3 group $xvars
// Using number of email reminders
* Alho1: common IMP across attempts and arms
alho logtotal3 group $xvars, repeats(nreminders)
* Alho2: common IMP across attempts but differs across arms
alho logtotal3 group $xvars, repeats(nreminders) impeq(group)
lincom [respond]groupXlogtotal3 + [respond]logtotal3 // IMP in intervention arm

## A.2. Allowing for incomplete intervention uptake

The following code is for quantitative compliance: results with binary compliance are obtained by redefining treat.

use DYDwide, clear
gen treat = min(pages1, 300) * (group==1) / 100
// Handling missing data by IPW
gen obs = !missing(logtotal3)
logit obs treat $xvars if group==1
predict pobs1
logit obs $xvars if group==1
predict pobs2
gen weight = pobs2/pobs1 if group==1
replace weight = 1 if group==0
ivregress 2sls logtotal3 $xvars (treat=group) [pw=weight], eform(Ratio)
// Handling missing data by ATR (Nagelkerke)
reg treat group $xvars
predict resid, resid
regress logtotal3 treat $xvars resid, eform(Ratio)
// Handling missing data by MI
use imputed2, clear
gen treat = min(pages1, 300) * (group==1) / 100
mim, category(fit): ivregress 2sls logtotal3 $xvars (treat=group), eform(Ratio)
mim, mcerror eform
